# Comparison of the rate of healthcare encounters for influenza from source-specific PM_2.5_ before and after tier 3 vehicle standards in New York state

**DOI:** 10.1038/s41370-024-00710-w

**Published:** 2024-08-10

**Authors:** Daniel P. Croft, Mark J. Utell, Philip K. Hopke, Han Liu, Shao Lin, Sally W. Thurston, Sathvik Thandra, Yunle Chen, Md Rayhanul Islam, Kelly Thevenet-Morrison, Carl J. Johnston, Tianming Zhao, Catherine Yount, David Q. Rich

**Affiliations:** 1 Pulmonary and Critical Care Division, Department of Medicine, University of Rochester Medical Center, Rochester, NY USA; 2 Department of Environmental Medicine, University of Rochester Medical Center, Rochester, NY USA; 3 Department of Public Health Sciences, University of Rochester Medical Center, Rochester, NY USA; 4 Institute for a Sustainable Environment, Clarkson University, Potsdam, NY USA; 5 Population Studies and Training Center, Brown University, Providence, RI USA; 6 Department of Environmental Health Sciences. University at Albany, the State University of New York, Albany, NY USA; 7 Department of Biostatistics and Computational Biology, University of Rochester Medical Center, Rochester, NY USA; 8 Department of Pediatrics, University of Rochester, Rochester, NY USA

**Keywords:** Source-specific PM_2.5_, Influenza, Traffic related air pollution, Accountability study, Air pollution, Biomass burning

## Abstract

**Background:**

Influenza healthcare encounters in adults associated with specific sources of PM_2.5_ is an area of active research.

**Objective:**

Following 2017 legislation requiring reductions in emissions from light-duty vehicles, we hypothesized a reduced rate of influenza healthcare encounters would be associated with concentrations of PM_2.5_ from traffic sources in the early implementation period of this regulation (2017–2019).

**Methods:**

We used the Statewide Planning and Research Cooperative System (SPARCS) to study adult patients hospitalized (*N* = 5328) or treated in the emergency department (*N* = 18,247) for influenza in New York State. Using a modified case-crossover design, we estimated the excess rate (ER) of influenza hospitalizations and emergency department visits associated with interquartile range increases in source-specific PM_2.5_ concentrations (e.g., spark-ignition emissions [GAS], biomass burning [BB], diesel [DIE]) in lag day(s) 0, 0–3 and 0–6. We then evaluated whether ERs differed after Tier 3 implementation (2017–2019) compared to the period prior to implementation (2014–2016).

**Results:**

Each interquartile range increase in DIE in lag days 0–6 was associated with a 21.3% increased rate of influenza hospitalization (95% CI: 6.9, 37.6) in the 2014–2016 period, and a 6.3% decreased rate (95% CI: −12.7, 0.5) in the 2017–2019 period. The GAS/influenza excess rates were larger in the 2017–2019 period than the 2014–2016 period for emergency department visits. We also observed a larger ER associated with increased BB in the 2017–2019 period compared to the 2014–2016 period.

**Impact statement:**

We present an accountability study on the impact of the early implementation period of the Tier 3 vehicle emission standards on the association between specific sources of PM_2.5_ air pollution on influenza healthcare encounters in New York State. We found that the association between gasoline emissions and influenza healthcare encounters did not lessen in magnitude between periods, possibly because the emissions standards were not yet fully implemented. The reduction in the rates of influenza healthcare encounters associated with diesel emissions may be reflective of past policies to reduce the toxicity of diesel emissions. Accountability studies can help policy makers and environmental scientists better understand the timing of pollution changes and associated health effects.

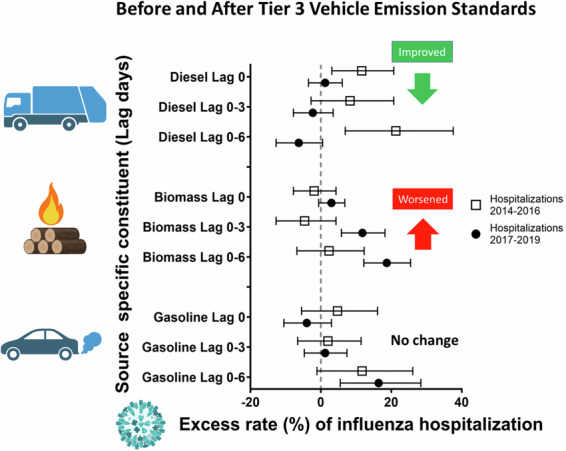

## Introduction

Influenza continues to be a concerning cause of adult morbidity and mortality each year in the United States since it represents the 13^th^ highest cause of death [1]. Although influenza rates were much lower during COVID-19, influenza remains a relevant seasonal respiratory infection as influenza rates have returned to pre-COVID rates after the acute phase of the COVID pandemic [2]. While the association between influenza and increased concentrations of PM_2.5_ has been reported by several groups previously [3–6], the association between constituent sources of PM_2.5_ and influenza is an area of active research. In our prior accountability study in New York State, we observed an increase in the relative rate of emergency department visits for influenza associated with an increased concentration of gasoline and diesel PM_2.5_ concentrations. This potential increase in the toxicity per unit mass in the setting of fuel regulations and decreasing particulate pollution highlighted the need for ongoing evaluation of complex health responses to atmospheric exposures [7].

As described previously [3, 8], a series of energy and vehicle related regulations were enacted including spanning coal-fired power plant closures, sulfur reduction in diesel for trucks, and now the more recent 2017 implementation of the tier 3 vehicle standards [9]. These Tier 3 standards, will reduce the average sulfur content of gasoline from 30 parts per million (ppm) to 10 ppm and also when fully phased in, will lead to a 70% reduction in the per-vehicle particulate matter standard for light to medium duty vehicle tailpipe emissions [10]. The updated Non-methane Organic Gases (NMOG) and NOx tailpipe emissions were set at 30 milligrams per mile (mg/mi) for model year 2025. Overall, the Tier 3 standards will match the California’s Super Ultra Low Emission Vehicle (SULEV) program so that the same cars can be sold in all 50 states in the U.S. [10].

In this study we again examined associations between influenza hospitalizations and emergency department visits associated with source-specific PM_2.5_ concentrations, specifically comparing these associations before and after Tier 3 vehicle emissions implementation on January 1, 2017.

While many PM_2.5_ constituents decreased from 2005 to 2013, secondary organic carbon (SOC) increased in the 2014–2016 period and has remained at relatively constant concentrations in the 2017–2019 period across all sites in New York State (NYS) [11]. Therefore, we also examined two forms of organic carbon, primary organic carbon (POC) and SOC. POC is directly emitted by a source and is commonly a combustion product while SOC is an atmospheric oxidation product of volatile organic compounds. The oxidative potential (ability to generate oxidative stress in the body) of SOC is greater than POC [12, 13]. The majority of PM_2.5_ is a combination of secondary inorganic aerosol (SIA; secondary nitrate [SN] and secondary sulfate [SS]) and secondary organic aerosols (SOA; e.g. secondary organic carbon). The fact that SOC concentrations did not fall in the 2017–2019 period raises the possibility of a sustained risk of influenza across periods due to SOC’s ability to disrupt the innate immune response to infection [14]. In our prior study of the difference in rates of influenza healthcare encounters associated with source-specific PM2.5 from 2005 to 2016 in adult New York State residents we observed an increase in the rate of healthcare encounters associated with influenza associated with interquartile range (IQR) increases in multiple source specific PM_2.5_ constituents including diesel, spark-ignition (gasoline) and biomass burning [7]. While SOC and POC were not included in this prior study the increased concentration of SOC and its potential health risk warranted the inclusion of both SOC and POC in the current study.

In this study, we examined whether the association between short term increases in ambient source-specific PM_2.5_ concentrations and influenza healthcare visits differed between the period prior to Tier 3 implementation (2014–2016) and the initial years of Tier 3 implementation (2017–2019). We hypothesized that the Tier 3 standards could result in a lower relative rate of hospitalization or emergency department visits for influenza associated with increased light-duty vehicle (gasoline), compression-ignition, heavy-duty diesel vehicle, and SOC PM_2.5_ concentrations in the 2017–2019 period compared to 2014–2016.

## Methods

### Study population

Respiratory infection hospital admissions and emergency department visits for adult New York residents (age > 18) were obtained from the Statewide Planning and Research Cooperative System (SPARCS) database. In total, *N* = 135,236 hospitalizations and *N* = 549,528 emergency department visits of adults living within 15 miles of the Buffalo, Rochester, Albany, Bronx, Manhattan, or Queens PM_2.5_ monitoring sites from January 1, 2014, to December 31, 2019, were retained. These monitoring sites were selected since they collect the PM_2.5_ samples for speciation analysis. We included participants with a primary diagnosis (at time of healthcare encounter) of influenza (ICD9 = 4870, 4871, 48811, 48812, 48881, 48882; ICD10 = J09X1, J09X2, J1000, J1001, J1008, J101, J1100, J1108 and J111. In the analysis, we paired pollution to the healthcare encounter in the same day (lag 0), prior 4 days (lag day 0–3) and prior 7 days (lag days 0–6). Given the vast majority (95%) of patients with influenza will become symptomatic within the first 2 days of infection [15], and then present for a healthcare encounter sometime in the next week (if at all), the 0–6 day lag period (7 days prior to presentation) may represent the exposure closest to the start of the influenza infection. While there is variability in when patients present for care after the start of symptoms, lag day 0 (same day) and lag days 0–3 are likely representative of exposure several days into influenza infection. For this reason, we focus our discussion on the associations at the 0–6 lag days (closest to the start of infection) and less so on the shorter lags. This study was approved by the Institutional Review Board at the University at Albany, State University of New York.

### Air pollution and meteorology measurements

We obtained PM_2.5_ compositional data from the U.S. Environmental Protection Agency (EPA) Chemical Speciation Network (www.epa.gov/aqs). In all six urban sites (Buffalo, Rochester, Albany, Manhattan, Bronx and Queens) daily samples were collected and analyzed every third day. Organic carbon, including primary organic carbon (POC) and secondary organic carbon (SOC), was measured every third day using thermo-optical analysis. The PM_2.5_ sources were identified using EPA positive matrix factorization (PMF) version 5. Further details on this approach can be found in our prior source-specific analyses by Squizzato et al. [16]. The seven PM_2.5_ sources included in this study included spark-ignition emissions (GAS), diesel (DIE), biomass burning (BB), road dust (RD), secondary nitrate (SN), secondary sulfate (SS), and pyrolyzed organic rich (OP). Daily ambient temperature and relative humidity were obtained from the National Weather Service.

### Statistical analysis

A time-stratified, case-crossover design [17, 18] was used to estimate the rates of respiratory infection hospital admissions and emergency department visits associated with each interquartile range increase in GAS concentrations on the same day (lag day 0). Assuming a common slope across sites for influenza hospital admissions for all sites, we fit a conditional logistic regression model stratified on each respiratory infection hospital admission matched set (1 case and 3-4 control periods per subject). This conditional logistic regression model regressed case–control status (i.e., case = 1, control = 0) against the mean GAS concentration on case and control days. Since the case-crossover approach controls for non-time-varying confounders, variables such as comorbidities, socioeconomic factors and seasons are all controlled for, by design. However, we included natural splines for temperature and relative humidity (4 degrees of freedom determined using Akaike’s information criterion) [19], and PM_2.5_ mass of all other sources (i.e., rPM2.5 (residual value) = PM_2.5_ – GAS). This same model was run for the mean GAS concentrations on lag days 0–3 and 0–6). From each model, we estimated the rate of hospitalizations or emergency department visits associated with each interquartile range increase in GAS concentration. Since we examined 3 lag times for GAS, statistical significance was defined as *p* < 0.017 (0.05/3), though we focused mainly on the magnitude and direction of the effect estimates rather than the statistical significance. Next, we examined whether the rates of respiratory infection admission associated with each interquartile range increase in GAS concentrations differed by period (2014–2016 and 2017–2019), by adding an interaction term (Period * GAS) to the model. We then re-ran these models for each of the other PM_2.5_ sources including biomass burning (BB), diesel (DIE), organic-rich phosphate (OP), road dust (RD), secondary nitrate (SN), secondary sulfate (SS) as well as SOC and POC concentrations. For each of these models a rPM_2.5_ was recalculated and included in the model. For example, the SOC rPM_2.5_ = PM_2.5_ – SOC and the RD rPM_2.5_ = PM_2.5_ – RD. By computing the residual for all sources, the rate of influenza healthcare encounters associated with a specific PM_2.5_ source (e.g. GAS) is thus independent of other of the PM_2.5_ mass from other specific sources (e.g. DIE, BB, SN) [20]. Similarly, the effect of SOC is independent of POC (but not the source-specific PM_2.5_ such as GAS and BB as these sources can contain SOC). All analyses were completed using R version 3.0.1 (https://www.r-project.org/).

## Results

Patients hospitalized for influenza were older (69 years old [SD of 18]) than patients treated and sent home from the emergency department (42 years old [SD of 18] (Table 1). The majority of patients hospitalized or treated in the emergency department were female (58% and 60% respectively) and sought care during the winter months (66% and 69% respectively). While the majority of hospitalized patients were White (62%), the majority of patients treated and sent home from the emergency department were Black (48%).

**Table 1:** Characteristics of respiratory infectious disease hospital admissions and emergency department visits (2014–2019), by study site/city.

	Hospitalizations (*N* = 20,205)		Emergency department visits (*N* = 70,855)	
Characteristic	*n*	%	*n*	%
Female	11,764	58	42,722	60
AGE in years: mean (standard deviation)	69 (18)		42 (18)	
≥18–64	7201	36	61,987	87
≥65	13,004	64	8868	13
Race/ethnicity				
White	9351	62	20,316	44
Black	4588	30	22,348	48
Native American	53	0	261	1
Asian	5122	6	7420	5
Native Hawaiian	42	0	165	0
Hispanic	3816	19	19,061	27
Year				
2014	2445	12	7809	11
2015	2150	11	4918	7
2016	2554	13	8360	12
2017	3015	15	7560	11
2018	5761	29	18,049	25
2019	4280	21	24,159	34
Season				
Spring	5978	30	18,398	26
Summer	215	1	626	1
Fall	609	3	2900	4
Winter	13403	66	48,931	69
Length of stay in days mean ± standard deviation	54.6 ± 7.0		0.2 ± 0.7	

### Pollution data

Using Theil-Sen nonparametric estimator and piecewise linear regression, our prior study [21] observed reduction in PM_2.5_ values across New York State ranging from 5.24% reduction in PM_2.5_ in Queens (95% CI: −5.61%, −4.39%) to a reduction of 2.51% in Albany (95% CI: −3.43%, −1.32%) from 2010 to 2019. When comparing the 2017–2019 period to the 2014–2016 period in our current study, the median concentration of GAS (2014–2016: 1.04 µg/m^3^; 2017–2019: 1.7 µg/m^3^) and SN (2014–2016: 1.3 µg/m^3^; 2017–2019: 1.8 µg/m^3^) showed the largest increases (Table 2). DIE (2014–2016: 0.49 µg/m^3^; 2017–2019: 0.54 µg/m^3^) and POC (2014–2016: 0.5 µg/m^3^; 2017–2019: 0.9 µg/m^3^) had smaller increases in concentrations between periods. We observed a decreased concentration of OP (2014–2016: 0.3 µg/m^3^; 2017–2019: 0.6 µg/m^3^), RD (2014–2016: 0.14 µg/m^3^; 2017–2019: 0.08 µg/m^3^) and SS (2014–2016: 0.9 µg/m^3^; 2017–2019: 0.3 µg/m^3^) in 2017–2019 compared to 2014–2016 and no appreciable change in BB or SOC.

**Table 2:** Distribution of source-specific PM_2.5_ concentrations (µg/m^3^), by time period, for case and control periods.

		Cases						Controls					
Site	Time Period	5^th^ %tile	25^th^ %tile	50^th^ %tile	75^th^ %tile	95^th^ %tile	Max.	5^th^ %tile	25^th^ %tile	50^th^ %tile	75^th^ %tile	95^th^ %tile	Max.
Total PM_2.5_	Overall	2.80	5.2	7.50	10.89	19.01	33.50	2.70	4.96	7.09	9.86	17.10	33.50
	2014–2016	2.80	5.6	8.10	11.52	18.60	33.50	2.80	5.30	7.60	10.50	17.80	33.50
	2017–2019	2.73	5.1	7.25	10.66	20.14	28.36	2.65	4.78	6.84	9.47	16.14	28.36
Biomass burning	Overall	−0.11	0.21	0.54	1.00	1.88	5.52	−0.11	0.19	0.50	0.93	1.75	5.52
	2014–2016	−0.02	0.22	0.52	0.98	1.79	5.28	−0.02	0.22	0.51	0.95	1.77	5.28
	2017–2019	−0.12	0.21	0.54	1.00	1.88	5.52	−0.12	0.17	0.50	0.91	1.75	5.52
Diesel	Overall	−0.02	0.31	0.52	0.80	1.35	2.69	−0.02	0.31	0.52	0.80	1.37	3.24
	2014–2016	0.03	0.30	0.49	0.75	1.26	2.69	0.01	0.30	0.49	0.73	1.19	3.24
	2017–2019	−0.03	0.31	0.55	0.82	1.42	2.35	−0.03	0.31	0.54	0.84	1.50	2.47
Gasoline	Overall	0.16	1.02	1.62	2.46	5.08	11.94	0.17	0.96	1.51	2.30	4.53	13.64
	2014–2016	−0.05	0.45	1.04	2.08	4.53	8.69	−0.06	0.41	1.00	1.97	4.10	13.64
	2017–2019	0.68	1.25	1.74	2.67	5.72	11.94	0.7	1.16	1.66	2.36	4.73	11.94
Organic-rich phosphate	Overall	−0.14	−0.07	0.13	0.50	1.40	13.05	−0.12	−0.06	0.14	0.50	1.39	14.67
	2014–2016	−0.12	−0.02	0.29	0.80	1.98	13.05	−0.12	−0.01	0.31	0.81	1.98	14.67
	2017–2019	−0.15	−0.08	0.06	0.42	1.22	3.49	−0.13	−0.07	0.08	0.42	1.06	4.09
Road dust	Overall	−0.04	0.03	0.10	0.27	0.73	5.68	−0.03	0.03	0.09	0.25	0.69	5.68
	2014–2016	−0.00	0.05	0.12	0.36	0.89	5.68	−0.01	0.06	0.14	0.34	0.88	5.68
	2017–2019	−0.07	0.02	0.08	0.23	0.61	4.81	−0.03	0.02	0.10	0.22	0.61	4.81
Secondary nitrate	Overall	0.06	0.72	1.63	3.41	6.73	14.55	0.05	0.59	1.53	2.81	6.57	14.55
	2014–2016	−0.01	0.46	1.31	2.94	6.60	14.55	−0.01	0.40	1.20	2.90	6.52	14.55
	2017–2019	0.11	0.90	1.80	3.52	6.73	11.33	0.09	0.73	1.59	2.81	6.73	12.47
Secondary sulfate	Overall	−0.64	−0.01	0.45	1.40	3.54	11.88	−0.61	−0.11	0.40	1.27	3.30	13.10
	2014–2016	−0.46	0.07	0.85	2.20	5.24	11.88	−0.46	0.06	0.90	2.23	5.14	13.10
	2017–2019	−0.70	−0.16	0.31	1.10	2.57	7.01	−0.64	−0.17	0.24	0.10	2.27	10.66
Primary organic carbon	Overall	0.10	0.39	0.78	1.19	2.00	3.72	0.10	0.37	0.72	1.16	1.70	4.21
	2014–2016	0.08	0.29	0.50	1.02	1.63	3.72	0.08	0.28	0.50	1.00	1.60	3.72
	2017–2019	0.11	0.50	0.94	1.24	2.17	3.38	0.10	0.47	0.90	1.20	1.74	4.21
Secondary organic carbon	Overall	0.01	0.36	0.67	1.13	2.24	7.05	0.01	0.33	0.63	1.10	2.17	7.05
	2014–2016	0.01	0.34	0.70	1.20	2.47	7.05	0.01	0.33	0.66	1.13	2.31	7.05
	2017–2019	0.01	0.37	0.67	1.12	2.22	4.60	−0.01	0.33	0.62	1.08	2.15	4.59
Temperature	Overall	−6.20	0.28	4.30	7.58	14.07	32.38	−6.70	0.21	4.07	7.60	14.31	33.09
	2014–2016	−6.60	−0.20	4.88	10.33	19.01	32.38	−6.60	−0.28	4.87	10.36	18.11	32.38
	2017–2019	−6.01	0.43	4.03	6.90	11.33	30.12	−7.01	0.28	3.92	6.86	11.94	33.09
Relative humidity	Overall	40.10	52.82	67.23	80.59	90.71	99.90	40.04	52.70	66.08	79.39	90.65	99.90
	2014–2016	37.80	48.90	62.42	74.11	87.38	95.06	37.13	49.21	62.43	74.11	86.64	95.06
	2017–2019	41.71	54.13	68.82	82.14	91.83	99.90	41.50	53.80	67.60	80.70	91.48	99.90

**Fig. 1:**
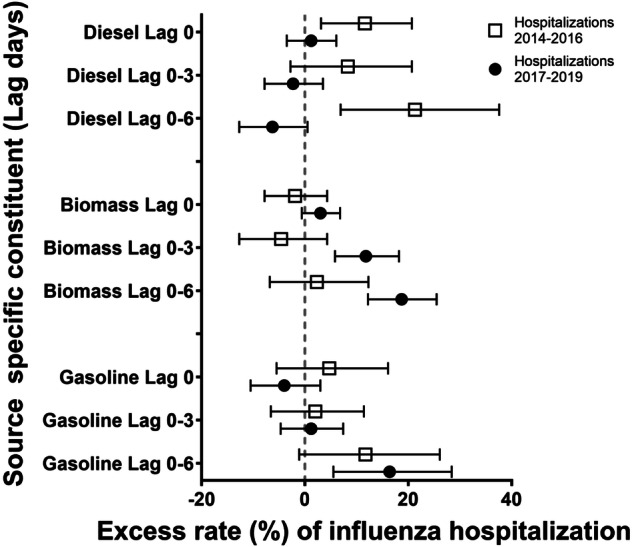
Excess rate of hospitalizations for influenza. Excess rate of hospitalizations for influenza associated with IQR increases in diesel (DIE), biomass burning (BB) and gasoline (GAS) at multiple lag times.

**Fig. 2:**
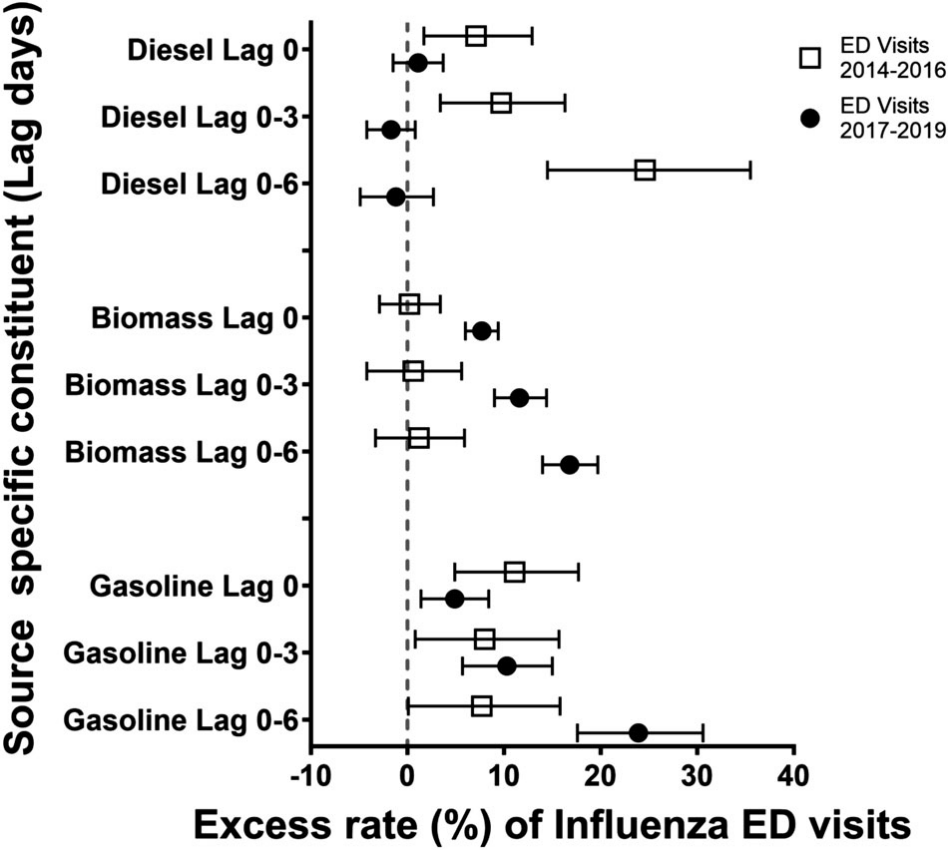
Excess rate of emergency department visits for influenza. Excess rate of emergency department visits for influenza associated with IQR increases in diesel (DIE), biomass burning (BB) and gasoline (GAS) at multiple lag times.

As hypothesized, the relative rates of hospital admission from influenza associated with interquartile range increases in DIE were smaller in the 2017–2019 period than the 2014–2016 period at all lag times. Specifically, each 0.3 μg/m^3^ increase in DIE concentration in the prior 6 days was associated with a 21.3% increased rate of influenza hospitalization (95% CI: 6.9, 37.6) in the 2014–2016 period, but a protective effect (−6.3% rate; 95% CI: −12.7, 0.5) in the 2017–2019 period (Table 3, Fig. 1). A similar pattern was observed for influenza emergency department visits and DIE in the prior 6 days. Specifically, each 0.4 μg/m^3^ increase in DIE concentration was associated with a 24.6% increased rate of emergency department visits for influenza (95% CI: 14.5, 35.5) in the 2014–2016 period, and a −1.2% rate (95% CI: −4.9, 2.7) in the 2017–2019 period (Table 4, Fig. 2).

**Table 3:** Excess rate of acute hospitalizations for influenza associated with each interquartile range increase in source-specific PM_2.5_.

	Lag day	0				0–3				0–6			
	Time period	*N*	IQR (µg/m^3^)	Excess rate (95% CI)	*p*-value*	*N*	IQR (µg/m^3^)	Excess rate (95% CI)	*p*-value*	*N*	IQR (µg/m^3^)	Excess rate (95% CI)	*p*-value*
Diesel (DIE)	2014–16	1853	0.4	11.6 (3.1, 20.7)	0.04	1513	0.3	8.3 (−2.8, 20.7)	0.09	1721	0.3	21.3 (6.9, 37.6)	<0.001
	2017–19	3475	0.4	1.2 (−3.5, 6.1)		3064	0.3	−2.3 (−7.8, 3.5)		3335	0.3	−6.3 (−12.7, 0.5)	
Biomass burning (BB)	2014–16	1853	0.5	−1.9 (−7.8, 4.3)	0.16	1513	0.5	−4.6 (−12.7, 4.3)	0.001	1721	0.4	2.3 (−6.8, 12.3)	0.004
	2017–19	3475	0.5	3.0 (−0.6, 6.8)		3064	0.5	11.8 (5.8, 18.2)		3335	0.4	18.7 (12.2, 25.5)	
Spark-ignition emissions (GAS)	2014–16	1853	2.2	4.7 (−5.5, 16.1)	0.13	1513	1.2	2.0 (−6.6, 11.4)	0.87	1721	1.5	11.7 (−1.1, 26.1)	0.54
	2017–19	3475	2.2	−4.0 (−10.5, 3.0)		3064	1.2	1.2 (−4.7, 7.4)		3335	1.5	16.4 (5.5, 28.4)	
Pyrolyzed organic rich (OP)	2014–16	1853	0.7	3.4 (−1.7, 8.8)	0.82	1513	0.7	5.7 (−1.3, 13.1)	0.77	1721	0.7	−8.9 (−17.0, −0.0)	0.01
	2017–19	3475	0.7	2.3 (−5.2, 10.4)		3064	0.7	7.8 (−3.8, 20.7)		3335	0.7	14.8 (0.3, 31.4)	
Road dust (RD)	2014–16	1853	0.2	1.5 (−2.2, 5.2)	0.6	1513	0.1	0.8 (−4.2, 6.1)	0.28	1721	0.1	1.5 (−3.5, 6.8)	0.55
	2017–19	3475	0.2	0.1 (−3.6, 3.9)		3064	0.1	−2.9 (−7.6, 2.0)		3335	0.1	−0.7 (−6.3, 5.1)	
Secondary nitrate (SN)	2014–16	1853	2	2.5 (−3.4, 8.7)	0.32	1513	1.9	1.2 (−7.0, 10.1)	0.83	1721	1.9	4.1 (−5.7, 14.9)	0.51
	2017–19	3475	2	−0.9 (−5.7, 4.2)		3064	1.9	0.2 (−6.3, 7.0)		3335	1.9	8.1 (−0.6, 17.5)	
Secondary sulfate (SS)	2014–16	1853	1.8	0.4 (−5.8, 7.0)	0.18	1513	1.3	1.0 (−6.8, 9.3)	0.21	1721	1.7	−13.7 (−23.9, −2.2)	0.36
	2017–19	3475	1.8	7.4 (−0.6, 16.1)		3064	1.3	8.8 (−0.4, 18.9)		3335	1.7	−6.2 (−17.6, 6.7)	
POC	2014–16	2075	0.5	0.5 (−5.1, 6.5)	0.69	1852	0.3	−1.3 (−7.3, 5.0)	0.26	2009	0.3	1.6 (−4.5, 8.0)	0.04
	2017–19	3588	0.5	−0.7 (−4.9, 3.6)		3174	0.3	2.6 (−2.4, 7.8)		3449	0.3	9.3 (3.7, 15.2)	
SOC	2014–16	2075	1	−2.5 (−9.9, 5.5)	0.23	1852	0.7	2.1 (−5.9, 10.7)	0.88	2009	0.7	−1.2 (−10.8, 9.4)	0.11
	2017–19	3588	1	−7.5 (−13.8, −0.7)		3174	0.7	1.4 (−6.0, 9.4)		3449	0.7	8.6 (−1.6, 19.9)	

^*^p-value for interaction between periods (2014–2016 and 2017–2019).

**Table 4:** Excess rate of acute emergency department visits for influenza associated with each interquartile range increase in source-specific PM_2.5_.

	Lag day	0				0–3				0–6			
	Time period	*N*	IQR (µg/m^3^)	Excess rate (95% CI)	*p*-value*	*N*	IQR (µg/m^3^)	Excess rate (95% CI)	*p*-value*	*N*	IQR (µg/m^3^)	Excess rate (95% CI)	*p*-value*
Diesel (DIE)	2014–16	4910	0.4	7.1 (1.7, 12.9)	0.05	3842	0.3	9.7 (3.4, 16.3)	<0.001	4653	0.4	24.6 (14.5, 35.5)	<0.001
	2017–19	13,337	0.4	1.1 (−1.5, 3.7)		11,485	0.3	−1.7 (−4.2, 0.8)		12,861	0.4	−1.2 (−4.9, 2.7)	
Biomass burning (BB)	2014–16	4910	0.4	0.2 (−2.9, 3.4)	<0.001	3842	0.4	0.6 (−4.2, 5.6)	<0.001	4653	0.4	1.2 (−3.3, 5.9)	<0.001
	2017–19	13,337	0.4	7.7 (6.0, 9.4)		11,485	0.4	11.6 (9.0, 14.4)		12,861	0.4	16.8 (14.0, 19.7)	
Spark-ignition emissions (GAS)	2014–16	4910	2.0	11.1 (4.9, 17.7)	0.06	3842	1.5	8.0 (0.8, 15.7)	0.56	4653	1.5	7.7 (0.1, 15.8)	<0.001
	2017–19	13,337	2.0	4.9 (1.4, 8.4)		11,485	1.5	10.3 (5.7, 15.0)		12,861	1.5	23.9 (17.6, 30.6)	
Pyrolyzed organic rich (OP)	2014–16	4910	0.7	−3.5 (−6.2, −0.7)	<0.001	3842	0.6	0.2 (−3.5, 4.0)	0.06	4653	0.7	−4.3 (−8.9, 0.6)	0.16
	2017–19	13,337	0.7	6.9 (3.4, 10.5)		11,485	0.6	6.5 (1.1, 12.1)		12,861	0.7	1.1 (−4.7, 7.3)	
Road dust (RD)	2014–16	4910	0.2	2.7 (0.5, 5.0)	0.00	3842	0.1	2.1 (−0.2, 4.4)	0.14	4653	0.1	0.9 (−1.7, 3.7)	0.40
	2017–19	13,337	0.2	−1.6 (−3.3, 0.2)		11,485	0.1	−0.0 (−1.8, 1.8)		12,861	0.1	−0.6 (−3.0, 1.9)	
Secondary nitrate (SN)	2014–16	4910	2.1	−2.9 (−6.6, 1.0)	0.01	3842	1.9	−3.4 (−8.4, 1.8)	0.04	4653	1.9	−3.4 (−9.2, 2.7)	<0.001
	2017–19	13,337	2.1	2.7 (0.1, 5.4)		11,485	1.9	2.3 (−1.2, 5.9)		12,861	1.9	12.9 (8.3, 17.8)	
Secondary sulfate (SS)	2014–16	4910	1.9	−2.6 (−6.5, 1.4)	<0.001	3842	1.3	−0.7 (−5.1, 4.0)	<0.001	4653	1.7	−11.3 (−17.7, −4.4)	<0.001
	2017–19	13,337	1.9	20.8 (16.1, 25.8)		11,485	1.3	15.4 (10.7, 20.3)		12,861	1.7	8.9 (1.7, 16.6)	
POC	2014–16	5644	0.4	0.7 (−2.6, 4.2)	0.07	4819	0.4	−2.9 (−6.8, 1.2)	<0.001	5509	0.4	−1.5 (−6.2, 3.3)	<0.001
	2017–19	13,879	0.4	4.1 (1.9, 6.3)		11,965	0.4	5.0 (2.1, 7.9)		13,295	0.4	11.8 (8.0, 15.6)	
SOC	2014–16	5644	0.8	−1.3 (−4.6, 2.1)	0.15	4819	0.6	−0.7 (−4.7, 3.4)	0.01	5509	0.7	−1.6 (−6.6, 3.7)	<0.001
	2017–19	13,879		1.4 (−1.5, 4.5)		11,965		5.0 (1.2, 8.9)		13,295		13.4 (7.7, 19.3)	

^*^p-value for interaction between periods (2014–2016 and 2017–2019).

The pattern for the association between GAS concentrations and influenza emergency department visits was different from DIE. Each 1.5 μg/m^3^ increase in GAS concentration was associated with a larger excess rate of influenza emergency department visits in the 2017–2019 period (ER = 23.9%; 95% CI: 17.6, 30.6) than in the 2014–2016 period (ER = 7.7%; 95% CI: 0.1, 15.8) for lag days 0–6, but not for lag day(s) 0 or 0–3 (where no differences were observed between periods) (Table 4, Fig. 2). The rate of influenza hospitalizations associated with each IQR increase in GAS concentration in lag days 0–6 was also larger in the 2017–2019 period (ER = 16.4%; 95% CI: 5.5%, 28.4%) than in the 2014–2016 period (ER = 11.7%; 95% CI: −1.1%, 26.1%).

The pattern of ER of biomass burning source-specific PM was the inverse of the DIE results, with no association between BB and influenza hospitalizations in the 2014–2016 period and increased relative rates in the 2017–2019 period at all lag periods. Specifically, each 0.4 μg/m^3^ increase in BB concentration in lag days 0–6 was associated with a 2.3% increased rate (95% CI: −6.8, 12.3) in the 2014–2016 period, and a 18.7% increased rate of hospitalizations for influenza (95% CI: 12.2, 25.5) in the 2017–2019 period (Table 3, Fig. 1). The pattern for BB/influenza emergency department visits was the same as hospitalizations. We observed a 1.2% increase in emergency department visits (95% CI: −3.3, 5.9) associated with each 0.4 μg/m^3^ increase in BB in the 2014–2016 period and a 16.8% increase (95% CI: 14.0, 19.7) in the 2017–2019 period (Table 4, Fig. 2).

The rate of influenza healthcare encounters associated with increased concentrations of SOC and POC were larger in the 2017–2019 compared to 2014–2016. Each 0.4 μg/m^3^ increase in POC concentrations in lag days 0–6 was associated with an 11.8% increase in emergency department visits for influenza (95% CI: 8.0, 15.6) in the 2017–2019 period, but only a 1.5% decrease in 2014–2016 (95% CI: −6.2, 3.3) (Table 4). Each 0.7 μg/m^3^ increase in SOC concentration in lag days 0–6 was associated with a 13.4% increase in emergency department visits for influenza (95% CI: 7.7, 19.3) in the 2017–2019 period and a 1.6% decrease (−6.6, 3.7) in 2014–2016. The difference in the rates between periods was slightly smaller for the hospitalizations for influenza compared to emergency department visits.

Similar to SOC and POC, the rate of influenza emergency department visits associated with SN and SS at the 0–6 lag time were larger in the 2017–2019 period compared to 2014–2016 period. One unique finding was the decreased rate of influenza hospitalizations associated with a 1.7 μg/m^3^ increase in SS at the 0–6 lag period (ER −13.7%; 95% CI: −23.9, −2.2). We observed larger rates of influenza hospitalizations associated with each 0.7 μg/m^3^ increase in OP concentration in lag days 0–6 in the 2017–2019 period (ER 14.8%; 95% CI: 0.3, 31.4) than the 2014–2016 period (ER of −8.9%; 95% CI: (−17.0, 0.0) at the 0–6 lag days (Table 3). There was no clear pattern of association for road dust and influenza healthcare encounters.

## Discussion

In this study of source-specific PM_2.5_ concentrations and influenza hospitalizations and emergency department visits in Rochester, NY, we estimated and compared the relative rate of influenza healthcare encounters associated with increased source-specific PM_2.5_, SOC, and POC concentrations both before (2014–2016) and after (2017–2019) Tier 3 vehicle emissions standards were implemented. As hypothesized, we observed a decrease in the rate of influenza hospitalizations and emergency department visits associated with increased DIE concentrations at all lag times in the 2017–2019 period compared to the 2014–2016 period. Unexpectedly, in the 2017–2019 period, we observed an increase in the rate of emergency department visits associated with spark-ignition/gasoline vehicle emissions (GAS) in the prior one week (lag days 0–6; compared to 2014–2016 period) and no change between periods at other lag times. A similar increase in the rate of healthcare encounters for influenza in the 2017–2019 period was observed for biomass burning, SS, POC, and SOC, but not other PM_2.5_ sources. The relative toxicity per unit mass of some of these PM_2.5_ sources may have been affected by implementation of the Tier 3 emissions standards and other prior emissions standards.

The need to examine the health effects of sources of PM_2.5_ is supported by our recent study on the effect of total PM_2.5_ on the rate of influenza healthcare encounters [22]. Despite reductions in overall PM_2.5_ concentrations during the early implementation period of the Tier 3 standards, the rates of influenza healthcare encounters associated with IQR increases in PM_2.5_ concentrations were not lower in the 2017–2019 period compared to the 2014–2016 period. As policy makers consider interventions to address specific sources of air pollution, the health effects of the remaining PM_2.5_ (which has a new relative composition) will require continued assessment.

Since the time of our prior study of source-specific PM_2.5_ and influenza [7] that reported increased rates of influenza hospitalization and emergency department visits associated with increased PM_2.5_, GAS, and DIE, the global COVID-19 pandemic redirected some of the environmental science community to focus environmental research on COVID-19 infection [23]. The uniqueness of the SARS-CoV-2 infection and its changing pandemic dynamics limited the applicability of the air pollution/COVID-19 studies to other seasonal respiratory viruses [24, 25]. Although there have been several studies estimating influenza/PM_2.5_ associations [4, 26], there have been few previous studies estimating associations between source-specific PM_2.5_ or PM_2.5_ constituents and influenza health care encounters [27]. In the interim from our prior source-specific study, a recent study in Guangdong Province, China observed a 2.8% increase in the relative risk of influenza infection associated with a 10 μg/m^3^ increase in PM_2.5_ concentrations in the 8 days prior to symptom onset [6]. Though only indirectly related to respiratory infection, an accountability study in Los Angeles, CA observed a decrease in risks of emergency department visits for asthma associated with increased PM_2.5_ constituents (organic carbon, sulfate, and nitrate) concentrations over time (2005–2016) [28]. Not all source-specific PM_2.5_ findings in our current study are consistent with these studies.

Our current study employed an improved PMF analysis to better apportion the aerosol resulting in differences between the effect estimates in the 2014–2016 period when comparing with our prior study source-specific/influenza study [7]. In this prior study we examined the association between increased source-specific PM_2.5_ (using the prior version of the PMF analysis) and influenza healthcare encounters in New York State from the same SPARCS database. Given the volume of data given the inclusion of three periods from 2005 to 2016 and the inclusion of culture negative pneumonia, we did not present the period-specific data in that study (only the total period). While the absolute values are not directly comparable, the trends in excess rates from 2014 to 2019 are important to compare. For example, the excess rates for GAS/influenza healthcare encounters decreased from 2005 to 2007 (ER 17%; 95% CI: 2.7, 33.3) to 2014–2016 (ER 6.1%; 95% CI: −2.4, 9.8) in lag days 0–6 (and similar pattern for lag days 0–3) (unpublished data). In the current study, rather than a trend of smaller excess rates over time, the excess rates for the GAS/influenza emergency department visit associations appeared similar (or larger) in the 2017–2019 period (ER = 23.9%; 95% CI: 17.6, 30.6) compared to the 2014–2016 period (ER 7.7%; 95% CI: 0.1, 15.8) for lag days 0–6.

The observation of a similar (or increased) relative rate for healthcare encounters for influenza associated with GAS in the 2017–2019 period compared to 2014–2016, despite the early implementation of the 2017 Tier 3 vehicle standards, is inconsistent with the hypothesis that Tier 3 vehicle standards could have reduced the toxicity per unit mass of the GAS component. Specifically, if the early implementation of the Tier 3 emissions standards decreased the secondary organic carbon (SOC) component of PM_2.5_, thereby reducing the oxidative potential of PM_2.5_, we would also expect to see a decrease in the rate of healthcare encounters for influenza associated with increased PM_2.5_ and GAS concentrations. The lack of reduction in the GAS/influenza association may be related to the incomplete fleet penetration of Tier 3 vehicles. While 36% of the current light duty vehicle fleet was purchased after 2017, we do not yet have data on what proportion of those vehicles met the Tier 3 standards (so the actual Tier 3 market penetration must be roughly 36% or lower) [29]. There may be other explanations including a change in the particle size distribution within GAS. The reduction in the mass of relatively less toxic particles such as secondary sulfate may have led to a relative increase in the toxicity per unit mass of the remaining sources. While the proportion of GAS consisting of UFP increased related in part to the 50% penetrance of gasoline direct injection in 2016, the change in toxicity related to this change will require further study.

In our prior study [7], the ERs for the DIE/Influenza emergency department visits were similar in the 2005-2007 period (ER 5.8% [95% CI: 2.6%, 9.0%]) compared to the 2014–2016 period (ER of 6.3% [95% CI 1.3%, 11.6%]) at the 0–6 lag days (unpublished data). In contrast, we observed a substantial decrease in the ER of the DIE/Influenza from 2014–2016 to the 2017–2019 period in the current study. The observed decrease in excess rates of influenza healthcare encounters associated with each 0.3–0.5 μg/m^3^ increase in DIE may be related to changes prior to the implementation of the 2017 Tier 3 vehicle standard. As a result of multiple diesel fuel related policies targeting the sulfur content of diesel, the concentrations of SO_2_ (and SS) have decreased [8]. Though NO_2_ decreased in part due to selective catalytic reduction (SCR) systems (required in new vehicles in 2010), the lack of reduction in SN concentrations may be related to an increase in ammonia that escapes the SCR system [30]. Since the Tier 3 vehicle emission standard predominantly addressed gasoline vehicles, it is unclear to what degree the early implementation of this regulation had the potential to reduce the toxicity per unit mass of DIE. Though not guaranteed, the toxicity of both DIE and GAS would be expected to continue to fall, as the emissions from both diesel and gasoline vehicles come below the recommended Tier 3 standard.

When considering non-traffic related PM_2.5_ sources, the increase in the rate of influenza healthcare encounters associated with BB concentrations in the 2017–2019 period, compared to the 2014–2016 period, may be related to a change in the characteristics of wildfire smoke or residential wood-burning smoke and/or their interaction with other pollutants in the outdoor atmosphere. While wildfire smoke has been suggested to comprise up to 25% of PM_2.5_ in the United States (and up to 50% in the Western U.S.) [31], the majority of BB comes from residential wood burning in the Rochester, NY area [32, 33]. The respiratory health effects of residential biomass burning are particularly difficult to study given the complexity of the factors that determine its emission profile including the condition of the fuel sources, efficiency of burning and ventilation of the indoor space [34]. It remains unclear which, if any, of these variables have changed in NYS to explain a change in relative toxicity in BB from residential wood burning. For wildfire smoke, climate change driven increases in outdoor temperature and reductions in humidity, combined with an increase in large diameter solid fuels (felled trees) due to high winds and draught or flooding is expected to lead to an increase in frequency, intensity and duration of wildfires [35]. Since the proportion of PM_2.5_ comprised of wildfire/biomass burning increases with further climate change, the reactive potential and toxicity per unit mass of PM_2.5_ may also increase [36, 37]. However, due to lower average temperature in New York State (NYS) compared to California, where the majority of the U.S. based literature focuses, we would expect less atmospheric reactivity, and potentially less toxicity, during studies similar to our current study in the winter months. Though wildfire smoke is not currently the dominant biomass source in NYS, the proportion of BB in NYS from wildfire smoke may increase in the future due to an expected increase in the frequency of dynamic weather patterns similar to the lingering low pressure system which led to significant biomass exposure in NYS from Canadian wildfires during the summer of 2023 [38]. Similar to the increased risk of hospitalization from asthma related to wildfire smoke, we may expect to see an increased risk of influenza associated with PM_2.5_ since the proportion of BB in NYS that comes from wildfire smoke increases [37].

Though the continued penetrance of Tier 3 vehicles into the New York State vehicle fleet is expected to result in a decrease in SOC [8], it is possible that the increase in organic carbon from wildfire activity over time will contribute more SOC to PM_2.5_ [39], thereby stabilizing SOC concentrations. The increase in rates of influenza healthcare encounters associated with increased SOC concentrations in 2017–2019 compared to 2014–2016 may indicate an increase in toxicity per unit mass of SOC. This pattern is the same as the main contributors to SOC, gasoline vehicle emissions (GAS) at lag days 0–6, and biomass burning. While we also observed an increased rate of influenza hospitalizations and emergency department visits with secondary sulfate (SS) (except for the 0–6 time period for hospitalization), due to the role of SS as a vector for a heterogeneous collection of condensates, the health effect of specific secondary sulfates remains uncertain [40]. Since the majority of PM_2.5_ is from inorganic and organic secondary aerosols (i.e. SIA and SOA), the reactivity of each constituent PM_2.5_ source such as traffic related air pollution (DIE and GAS) and biomass burning have been found to be more similar to the higher oxidative potential of SOC than the less reactive POC [12, 41]. For a viral infection like influenza, PM_2.5_ constituents with high oxidate potential (e.g. DIE, GAS and BB) have the potential to enhance viral entry into cells, impair pathogen recognition, immune signaling, and immune cell function [14].We will need to continue to focus future studies on the potential pathophysiologic mechanisms of the association between PM_2.5_ and influenza healthcare encounters and what collection of sources appear to be the main drivers of this association.

Inequity in both exposure to PM_2.5_ and treatment for influenza exist in the U.S. Our study observed that a higher proportion of Black and Hispanic patients with influenza were treated and released from the ED (48% and 27% respectively) compared to those who were admitted (30% and 19% respectively). Our patient proportions are not consistent with national data observing the odds of hospitalization (including intensive care) and death for influenza are higher in Black, Hispanic, and Asian patients when compared to white patients [42]. It is also well known that Black and Hispanic communities suffer a disproportionate burden of traffic related air pollution [43]. These two facts combine to increase the risk of a poor outcome from influenza infection in Black and Hispanic communities when compared to White communities. In a future analysis, we will need to consider how the difference in our local healthcare practice patterns and geographic distribution of source-specific PM_2.5_ may affect the association between PM/influenza in different racial and ethnic communities.

Although there were several strengths of this study, including a large sample size and inclusion of the 4 largest cities in New York State, there are several limitations that should be considered when making inference. First, there is likely exposure misclassification and downward bias resulting from assignment of the same air pollutant concentrations for everyone within 15 miles of an individual monitor (e.g., Buffalo), no matter how far they live from the monitoring station. Second, this study was a wintertime study given the seasonality of influenza infection, limiting the generalizability to year-round respiratory infections or bacterial respiratory infections. Last, to be consistent with our previous analyses, we used a case-crossover design and conditional logistic regression analyses to estimate the rate of influenza hospitalizations and emergency department visits associated with source-specific PM_2.5_, SOC, and POC concentrations in the previous 7 days. However, as described in our previous analysis of influenza/PM_2.5_ associations in these same cities, we cannot assess lag periods longer than 7 days without overlap of case and control period dates and thus case and control period pollutant concentrations [22]. Thus, we are limited to assessment of pollutant concentrations in just the previous 1–7 days. Last, the study population is only adult residents, and not children, and only those adults living in urban centers of the state (i.e., within 15 miles). Thus, generalizability of results to children or to adult residents in rural locations is less certain.

## Conclusion

While there did not appear to be any clear reduction in influenza healthcare encounters from gasoline emissions during the early implementation period of the Tier 3 emissions standards, we did see a reduction in the rate of healthcare encounters associated with diesel emissions. It is possible that we are seeing the effect of prior diesel regulations to lower sulfur content and that the ability to fully evaluate the health effects of the gasoline emission regulation may come only later once the Tier 3 vehicle penetration into the entire New York State vehicle fleet increases. The finding of a potential increase in the toxicity per unit mass of biomass burning (including wildfires) is concerning due to the expectation that this component of PM_2.5_ is expected to continue to increase as a proportion of PM_2.5_ due to climate change. Furthermore, addressing the burden of both influenza and air pollution on disproportionately affected communities will be an important research focus in the future, especially as climate change serves to worsen air quality and increase the frequency and intensity of natural disasters. Given the complexity of the atmospheric chemistry of a changing PM_2.5_ composition, future studies will need to evaluate effect modification of the rate of respiratory infection healthcare encounters associated with PM_2.5_ by heat waves and cold waves or implementation of air quality policies.

## Data Availability

The data that support the findings of this study are available from the Statewide Planning and Research Cooperative System (SPARCS) in New York State, but restrictions apply to the availability of these data, which were used under an agreement for the current study, and so are not publicly available. Data can be made available from SPARCS after an application process https://www.health.ny.gov/statistics/sparcs/.
